# Efficacy and safety of apatinib monotherapy for patients with advanced breast cancer: a systematic review and meta-analysis

**DOI:** 10.3389/fonc.2022.940171

**Published:** 2022-08-01

**Authors:** Xuchen Huang, Xuhua Hu, Tongbo Yi

**Affiliations:** ^1^ Department of Thyroid and Breast Surgery, Taizhou People’s Hospital Affiliated to Nanjing Medical University, Taizhou, China; ^2^ The Second Department of General Surgery, Hebei Medical University Fourth Affiliated Hospital, Shijiazhuang, China

**Keywords:** apatinib, locally advanced breast, metastatic breast cancer, tyrosine kinase inhibitor, meta-analysis

## Abstract

**Background:**

Patients with advanced breast cancer usually have poor prognosis. Apatinib is a small-molecule tyrosine kinase inhibitor, and the reports regarding the efficacy and safety of apatinib monotherapy for advanced breast cancer in the current literature are controversial. Therefore, we performed a systematic review and meta-analysis to collect and pool efficacy and safety data of apatinib monotherapy for advanced breast cancer with the aim of providing up-to-date evidence to aid clinical practice.

**Methods:**

This study was registered at PROSPERO (CRD42020190049). Three literature databases, including PubMed, EMBASE, and Cochrane Library, were searched. For evaluating efficacy, the objective response rate and disease control rate were extracted or calculated. Safety was evaluated in terms of the proportions of patients with grade 3 or 4 treatment-related adverse events. The pooled proportions of the outcomes and their 95% confidence interval were shown. The Kaplan–Meier curves of overall survival and progression-free survival were pooled from the extracted data of the included studies. Furthermore, pooled medians for overall survival and progression-free survival were calculated. A *p*-value of < 0.05 was considered statistically significant.

**Results:**

Six studies were included and deemed eligible for further quality evaluation and analysis. The pooled objective response rate and disease control rate were 20.4% and 71.6%, respectively. The pooled proportions of four hematologic adverse events ranged from 2.6% to 6.9%. The pooled proportions of hypertension, hand-foot syndrome, transaminase increased, and proteinuria ranged from 4.1% to 24.3%, and other non-hematologic adverse events were <1%. The pooled median progression-free survival and overall survival were 4.00 and 10.43 months, respectively, in cases of advanced breast cancer treated with apatinib.

**Conclusions:**

This study confirms the reliable efficacy of apatinib monotherapy for advanced breast cancer. However, non-hematologic grade 3–4 adverse events, especially hypertension, are more frequently observed during apatinib treatment than during treatment with other tyrosine kinase inhibitors, such as sunitinib or sorafenib.

**Systematic Review Registration:**

https://www.crd.york.ac.uk/prospero/, identifier CRD42020190049.

## Introduction

Breast cancer (BC) is the most frequently diagnosed cancer among women (1). With significant improvements in treatment efficacy, the overall survival (OS) of patients has been prolonged, with the exception of patients with locally advanced BC (LABC) or metastatic BC (MBC) (2). Consequently, patients suffering from advanced BC (ABC) usually have a poor prognosis. Furthermore, resistance to traditional chemotherapy and the restricted performance status of older patients underline the importance of introducing effective drugs with limited toxicity in clinical practice. This is especially urgent for advanced triple-negative BC (TNBC), which is more aggressive and with relatively poorer prognosis, but lacks of expression of classical therapeutic targets, including estrogen receptor (ER), progesterone receptor (PR), and human epidermal growth factor receptor 2 (HER2) (3).

Tumor angiogenesis, recognized as one of the “hallmarks of cancer”, plays an essential role in tumor growth and metastasis, which requires endothelial cell proliferation and migration (4). Vascular endothelium growth factor (VEGF) can promote endothelial cell proliferation and migration through their receptors, vascular endothelial growth factor receptor 1, 2, and 3 (VEGFR-1, -2 and -3), and is thus of great importance in tumor angiogenesis. Hence, VEGFR inhibition can be a potential anti-angiogenic therapy. In particular, high-affinity VEGFR-2 is mainly expressed in vascular endothelial cells and is the major mediator of the functional effects of VEGF (5, 6).

Apatinib is a small-molecule tyrosine kinase inhibitor (TKI) that selectively binds to and inhibits VEGFR-2 (7). In 2014, two phase II clinical trials demonstrated the objective efficacy results and acceptable toxicity in pretreated metastatic TNBC and non-TNBC patients (8, 9). These two trials showed that the objective response rates (ORRs) were 16.7% and 17.9%, which were lower than other commonly used single-agent drugs for patients with ABC such as paclitaxel, capecitabine, and gemcitabine (10–13). These results limited physicians’ interest in apatinib’s application, and there were few studies comparing efficacy of apatinib monotherapy with guideline-accepted single-agent drugs. Apatinib monotherapy for patients with ABC is primarily considered following the failure of standard chemotherapy regimens or intolerance to chemotherapy. However, in more recent real-world studies, the ORR can rise up to 41.7% in patients with ABC (14). Hence, the up-to-date results based on existing studies are necessary for evidence-based clinical references. These results can provide evidence for further clinical trials comparing apatinib with other widely used drugs in different clinical settings. We design this systematic review and meta-analysis to collect and pool efficacy and safety data of apatinib monotherapy for ABC and present the following article in accordance with the Preferred Reporting Items for Systematic Reviews and Meta-Analyses (PRISMA) reporting checklist.

## Methods

A workflow diagram showing the study design is presented in **Supplementary Figure 1**.

### Search strategy

This study was registered at PROSPERO (registration number CRD42020190049). Three literature databases (PubMed, EMBASE, and Cochrane library) were searched from inception to 21 March 2022. The following medical subject headings or key words were used: (“Breast Cancer” or “Breast Neoplasm” or “Breast Malignant Tumor” or “Breast Carcinoma” or “Mammary Cancer” or “Mammary Carcinoma” or “Mammary Neoplasm”) and (“apatinib” or “YN968D1” or “rivoceranib”). There were no specific restrictions regarding publication language or study design; however, all potentially eligible studies underwent manual screening with predetermined inclusion and exclusion criteria. The references included in the key studies and reviews were also screened for eligibility.

### Study selection

Two authors independently performed study selection, quality assessment, and data extraction using predefined forms. Discrepant data were reviewed and resolved by discussion with the third author.

Studies should fulfill the following population, intervention, comparison, outcomes, and study design criteria. Population: The patient population of interest suffered from histologically confirmed LABC or MBC. Patients were all ≥ 18 years old and had at least one measurable lesion. The molecular subtypes of BC were not limited. Patients had Eastern Cooperative Oncology Group (ECOG) performance status ≤ 3. Intervention: All included patients failed in at least first-line chemotherapy regimen for ABC or were beyond lines of standard therapy. They were treated with apatinib monotherapy orally with the initial doses decided by physicians during the study periods. Comparison: In potentially included studies, the efficacy of apatinib monotherapy can be compared with other treatment groups. However, our study only focuses on the efficacy and safety of apatinib monotherapy, comparison groups were not considered, and only groups including patients with ABC administrated with apatinib monotherapy were included. Outcomes: Included studies should report at least one clinical outcome of interest predetermined in our study. These clinical outcomes included two aspects: the efficacy and safety of apatinib monotherapy during study duration. Efficacy evaluation included ORR, disease control rate (DCR), and survival results. Safety evaluation included treatment-related adverse events (AEs) and toxicity-related dose change rates. Study design: All prospective and retrospective studies published in English or Chinese were potentially included in our study.

Studies with less than 10 efficacy-evaluable patients were excluded. Among the studies with similar inclusion criteria and overlapping patient populations derived from the same research center, only the study with the latest or largest population was included. Patients were excluded if additional treatments were administrated besides apatinib or patients were contraindicated for use of apatinib, including hematologic and coagulation disorders, severe hypertension, inadequate hepatic, renal, and cardiac functions. Case reports, reviews, editorials, letters, meta-analysis, and preclinical studies were also excluded. The study selection process was summarized using a PRISMA flow diagram.

### Quality assessment

For the assessment of methodological quality of single-arm studies, the methodological index for non-randomized studies (MINORS) was employed. MINORS comprises 12 items, the first eight of which are designed for non-comparative, non-randomized controlled trials :(1) a clearly stated aim, (2) inclusion of consecutive patients, (3) prospective collection of data, (4) endpoints appropriate to the aim of the study, (5) unbiased assessment of the study endpoint, (6) follow-up period appropriate to the aim of the study, (7) loss to follow-up less than 5%, and (8) prospective calculation of the study size. Items were scored 0, 1, or 2 for without report, inadequate report, and adequate report, respectively. A study could earn a maximum score of 16 points, and studies scoring ≥10 were considered eligible for further analysis.

### Data extraction

For each included study, the following data were extracted and recorded: first author, publication year, study design, number and median age of patients, patient population (TNBC, non-TNBC), starting dose of apatinib, and follow-up duration. To evaluate efficacy, ORR and DCR were extracted or calculated in terms of the proportion of patients who achieved complete response (CR) or partial response (PR), and the proportion of patients with CR, PR, or stable disease. Toxicity was evaluated by the proportion of patients with grade 3 and 4 treatment-related AEs based on Common Terminology Criteria for Adverse Events. These AEs included hematologic (leukopenia, neutropenia, anemia, and thrombocytopenia) and non-hematologic (hypertension, hand-foot syndrome, proteinuria, bilirubin increased, transaminase increased, diarrhea, and vomiting) events. In addition, the proportions of patients who experienced treatment interruption, dose reduction, and treatment discontinuation were also calculated.

### Data analysis and statistical methods

Analyses were performed using packages of meta (15), digitize (16), and metaSurvival (17) for R software (18). The proportions of outcomes from eligible studies were calculated and pooled. Freeman-Tukey double arcsine transformation of original proportions was performed for variance stabilization before pooling, and back-transformed proportions were calculated to facilitate the interpretation of the outcomes. A random-effect model was applied to calculate the pooled proportion and its 95% confidence interval (CI) in cases of significant heterogeneity among studies. Otherwise, the fixed-effect model was used. A p-value of <0.1 for Cochran’s Q statistic test or I^2^ >50% was regarded as indicating significant heterogeneity among the studies. Moreover, the potential publication bias of the included studies was examined using Egger’s test if more than 10 studies were included. The Kaplan–Meier curves of OS and progression-free survival (PFS) were pooled with the data from the included studies, and the pooled median of OS and PFS were calculated as appropriate (19). A *p*-value of <0.05 was considered statistically significant.

## Results

### Search results

**Figure 1:**
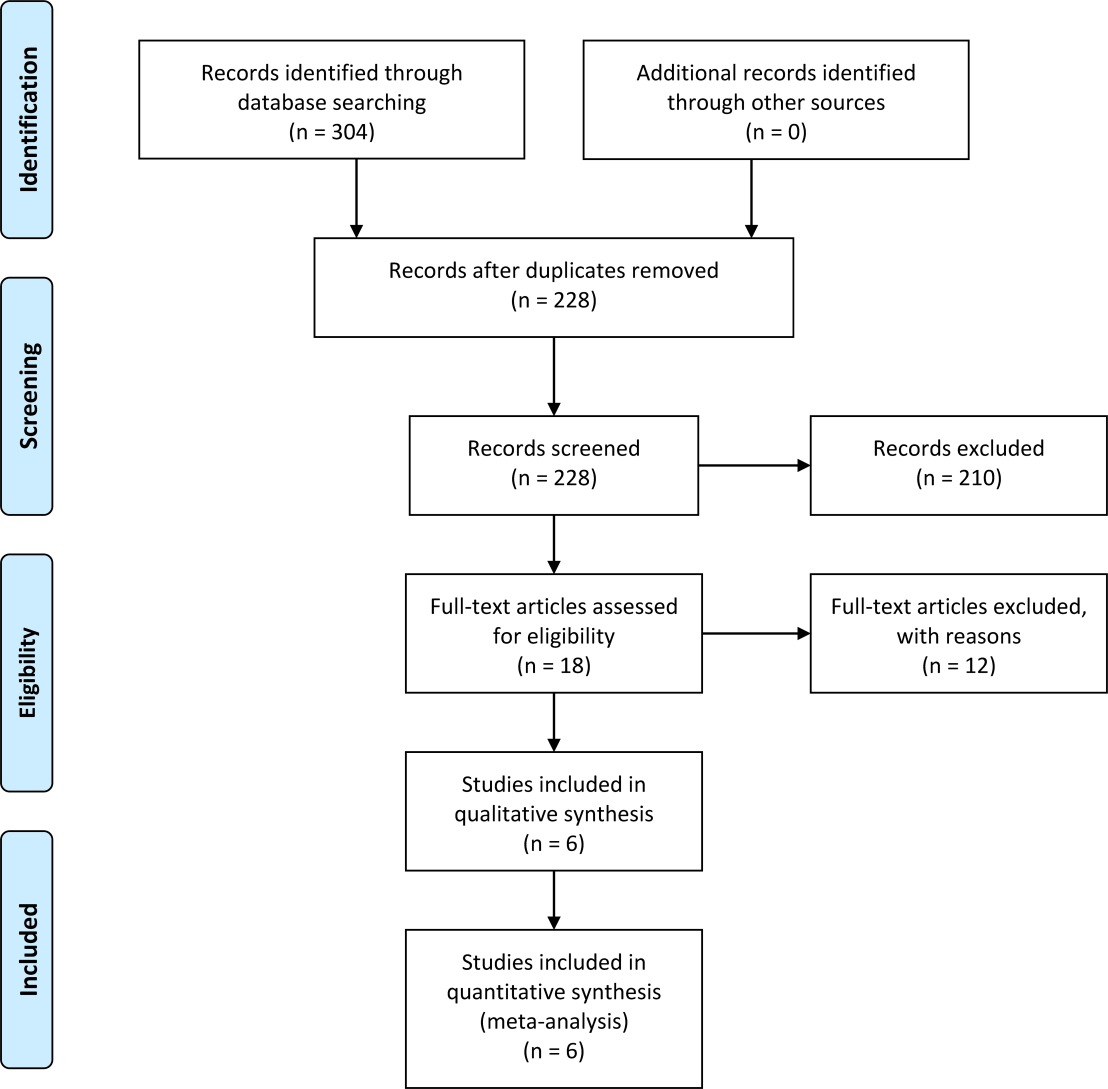
Flow chart of the selection process of the studies.

From the three databases that were searched, 304 studies were retrieved; 78 from PubMed, 193 from EMBASE, and 33 from Cochrane Library. **Figure 1** summarizes the selection process of the studies. After removing duplicates and screening titles or abstracts, 18 articles underwent full text evaluation. Following this, 12 studies were excluded for the following reasons: less than 10 patients for efficacy evaluation (n = 6) (20–25), unable to extract outcomes of interest (n = 1) (26), overlapping patient populations (n = 1) (27), and conference abstracts (whose results were also published in original research articles) (n = 4) (28–31). Among the remaining six studies, one study administered two patients with apatinib and trastuzumab and included one patient with ECOG performance status ≥ 2 and another patient unable to evaluate performance status. We still included this study because the proportion of patients without meeting the inclusion criteria was low. Finally, six studies were included and deemed eligible for further quality evaluation and analysis (7–9, 14, 32, 33).

### Quality assessment

The results of MINORS for methodological quality evaluation are summarized in **Table 1**. The MINORS scores ranged from 10 to 15, with the major drawbacks of the retrospective studies being the lack of prospective study size calculation and data collection. Moreover, although two retrospective studies were designed to evaluate the efficacy and safety of apatinib in patients with ABC, the authors emphasized more on reporting combined patient populations administered with apatinib monotherapy or with extra treatments, which was inconsistent with the study’s aim and, hence, led to decreased attention toward patients treated with apatinib monotherapy.

**Table 1:** Methodological quality evaluation of the included studies using methodological index for non-randomized studies.

Study ID	A clearly stated aim	Inclusion of consecutive patients	Prospective collection of data	Endpoints appropriate to the aim of the study	Unbiased assessment of the study endpoint	Follow-up period appropriate to the aim of the study	Loss to follow up less than 5%	Prospective calculation of the study size
Hu2014(1) (8)	2	2	2	2	1	2	2	2
Hu2014(2) (9)	2	2	2	2	1	2	2	2
Lin2017 (32)	2	2	0	2	1	2	2	1
Liu2021 (7)	1	2	0	1	1	2	2	1
Lü2018 (14)	2	2	0	2	1	2	2	1
Shen2019 (33)	1	2	0	1	1	2	2	1

The prospective studies possessed high methodological quality, although they did not adequately report the methods for unbiasedly assessing endpoints.

### Data extraction

The abovementioned outcomes of interest and clinical characteristics of the included studies were extracted. The study characteristics and outcomes of efficacy evaluation are shown in **Table 2**. A total of 237 patients were included in the analysis, with median ages ranging from 44 to 52.5 years. Among the final six studies, Hu et al. conducted two phase II trials on patients suffering from non-TNBC and TNBC in 2014 (8, 9). To distinguish between these two trials, we assigned them the study IDs of Hu2014 (1) (8) and Hu2014 (2) (9), respectively. Although some studies reported certain outcomes of interest, we only included data from the patient population treated with apatinib monotherapy, whereas data from studies using combined patient populations administered with apatinib monotherapy and apatinib with other drugs were not included.

**Table 2:** Outcomes of interest extracted from the included studies.

Study ID	Study design	Population (TNBC/non-TNBC)	Starting dose (mg daily)	Sample size	Median age (years)	Median follow-up duration (months)	ORR	DCR
Hu2014(1) (8)	Prospective	Non-TNBC	500	38	49	10.1	6/36	24/36
Hu2014(2) (9)	Prospective	TNBC	750	25	51	30.8	8/22	17/22
			500	59	52	19.7	6/56	14/56
Lin2017 (32)	Retrospective	Both	250/500/750	52	52.5	NA	10/45	31/45
Lü2018 (14)	Retrospective	TNBC	500	14	44	7.2	7/14	20/24
		Non-TNBC		10			3/10	
Shen2019 (33)	Retrospective	Both	500	17	NA	NA	2/17	12/17
Liu2021 (7)	Retrospective	Both	250/425/500/850	22	NA	NA	NA	NA

TNBC, triple-negative breast cancer; ORR, objective response rate; DCR, disease control rate; NA, not available for patients with apatinib monotherapy.

### Efficacy evaluation

#### ORR and DCR of apatinib monotherapy for ABC

**Figure 2:**
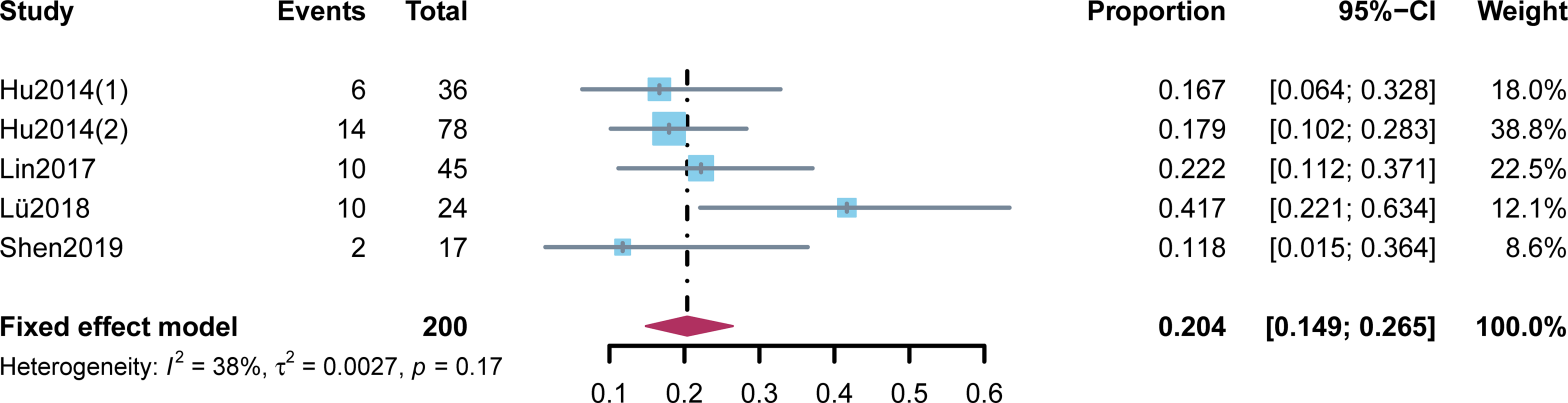
Meta-analysis of the objective response rates.

**Figure 3:**
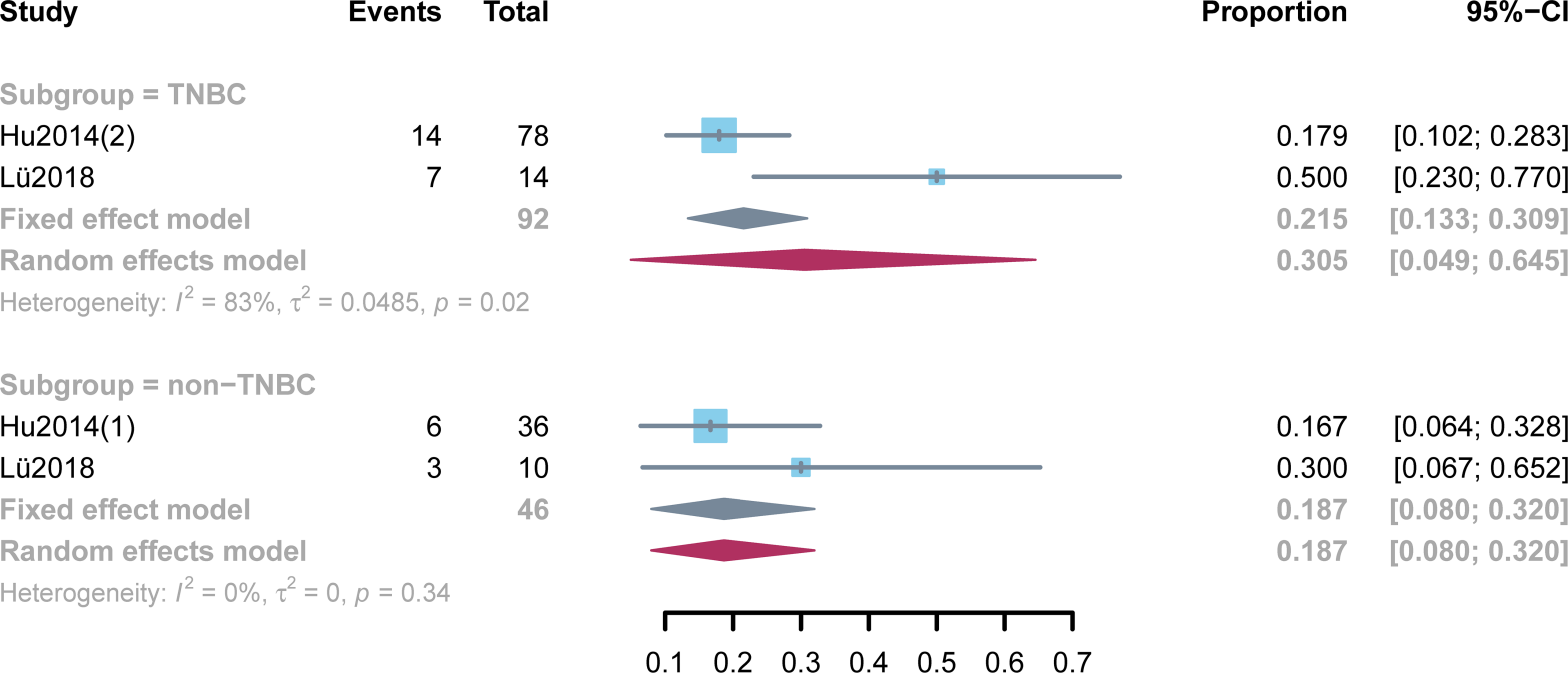
Meta-analysis of objective response rates with subgroup analysis. **TNBC,** triple-negative breast cancer**; non-TNBC,** non–triple-negative breast cancer.

Five studies reported ORRs ranging from 11.8% to 41.7% (**Figure 2**). High heterogeneity was not found among the studies (*p* = 0.17, I^2^ = 38%). Two hundred patients were included into meta-analysis, and the pooled ORR was 20.4% (95% CI: 14.9%–26.5%). A subgroup analysis was performed on the basis of the molecular subtype of BC (**Figure 3**). Three studies provided more detailed ORRs based on TNBC or non-TNBC. High heterogeneity was found among the studies that included patients with TNBC, for which a random-effect model was used, whereas a fixed-effect model was applied when pooling the results of patients with non-TNBC (I^2^ = 83% and I^2^ = 0%, respectively). A higher ORR was found in TNBC than in non-TNBC (30.5% and 18.7%, respectively).

**Figure 4:**
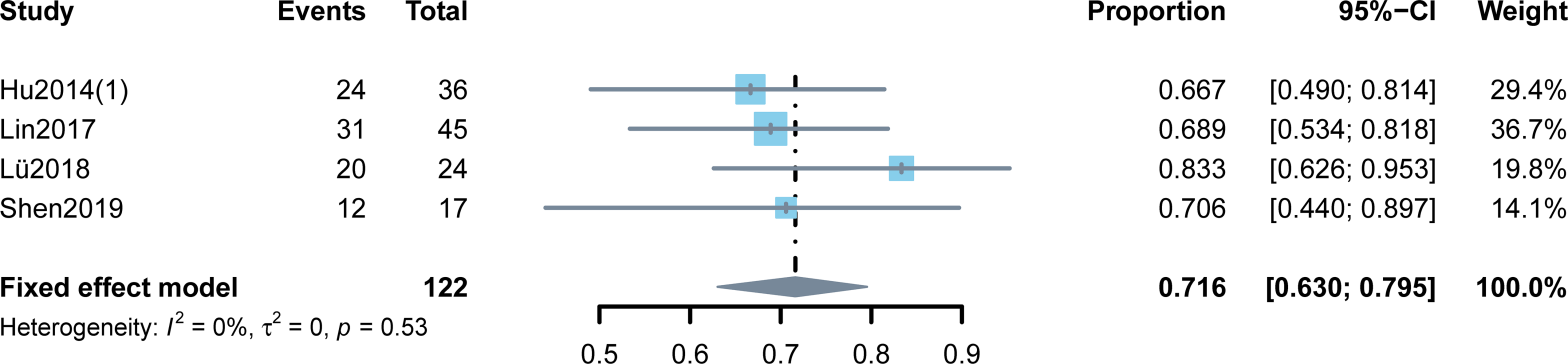
Meta-analysis of the disease control rates.

When pooling the proportions of DCR of these five studies, high heterogeneity was observed (I^2^ = 81%). Most of these five studies reported a mixed DCR for patients with TNBC and non-TNBC, so a subgroup analysis based on the molecular type of BC was not performed. Sensitive analysis was used to explore the sources of heterogeneity, and a high heterogeneity was found in Hu2014 (2) (9). Hence, Hu2014 (2) (9) was omitted and the pooled DCR following that was found to be 71.6% (95% CI: 63.0%–79.5%) (**Figure 4**).

Four studies provided ORRs and DCRs of patients receiving apatinib with a starting dose of 500 mg/day, which is the most common dose for patients. These patients had ORRs ranging from 10.7% to 41.7% and DCRs from 25% to 83.3%, respectively (8, 9, 14, 33). The pooled ORR and DCR were 18.6% and 60.9%, respectively (**Supplementary Figures 2**, **3**). In the study where patients with ABC received a starting dose of apatinib 750 mg/day, the ORR and DCR was 36.4% and 77.3%, respectively (9), which are higher than the pooled results in patients receiving a starting dose of 500 mg/day.

#### Pooling Kaplan–Meier curves of PFS and OS

Four and three studies provided Kaplan–Meier curves of PFS and OS, respectively, from the patients administered with apatinib, which were suitable for data extraction and pooling of Kaplan–Meier curves. The pooled Kaplan–Meier curves are shown in **Supplementary Figures 4**, **5**. The pooled median PFS and OS were 4.00 and 10.43 months, respectively, for patients with ABC treated with apatinib monotherapy.

### Safety evaluation

#### Hematologic toxicity ≥ grade 3

Four studies reported hematologic toxicity. Four hematologic ≥ grade 3 AEs were selected for toxicity evaluation, including thrombocytopenia, leukopenia, neutropenia, and anemia. The pooled proportions of these four hematologic AEs ranged from 2.6% to 6.9%. The detailed results are shown in descending order in **Table 3**, with only thrombocytopenia pooling showing high heterogeneity among the studies. A random-effect model was used.

**Table 3:** Pooled analysis of adverse events (grades 3–4) and dosage adjustment.

Adverse events or dose adjustment types	Outcomes	Number of studies	Events/total number of patients	Pooled proportions (%)	95% CI (%)
Hematologic toxicity ≥ Grade 3	Thrombocytopenia	4	18/198	6.9	1.3 to 15.4
	Leukopenia	3	9/160	5.5	2.2 to 9.9
	Neutropenia	3	6/174	3.4	0.9 to 6.9
	Anemia	3	5/160	2.6	0.4 to 6.1
Non-hematologic toxicity ≥ Grade 3	Hypertension	4	49/198	24.3	18.5 to 30.7
	Hand-foot syndrome	5	31/215	13.4	8.9 to 18.5
	Proteinuria	5	22/215	9.3	5.5 to 13.8
	Transaminase increased	4	6/139	4.1	1.1 to 8.5
	Bilirubin increased	4	2/198	0.5	0.0 to 2.5
	Vomiting	4	2/191	0.6	0.0 to 2.7
	Diarrhea	3	1/112	0.4	0.0 to 3.4
Dose adjustment	Treatment interruption	4	102/198	56.4	39.9 to 72.3
	Dose reduction	4	86/198	43.3	36.4 to 50.5
	Treatment discontinuation	3	14/114	12.1	6.4 to 19.0

#### Non-hematologic toxicity ≥ grade 3

Six studies reported non-hematologic toxicity, and seven non-hematologic ≥ grade 3 AEs were selected for evaluation, including hypertension, hand-foot syndrome, proteinuria, bilirubin increased, transaminase increased, diarrhea, and vomiting. High heterogeneity was not found upon pooling these seven non-hematologic AEs. The pooled proportions of bilirubin increased, diarrhea, and vomiting were < 1%. The pooled proportions of grade 3–4 hypertension, hand-foot syndrome, transaminase increased, and proteinuria ranged from 4.1% to 24.3%. All the seven pooled proportions are shown in descending order in **Table 3**.

#### Toxicity-related dose adjustment

Four studies reported toxicity-related dose adjustment. The pooled proportions of patients that underwent dose reduction and treatment discontinuation are shown in descending order in **Table 3**. A high heterogeneity was found when pooling the proportions of treatment interruption (I^2^ = 81%). A random-effect model was used.

### Publication bias assessment

Publication bias assessment was not performed due to the limited number of studies included.

## Discussion

Apatinib was initially approved for use in China for patients with previously treated advanced adenocarcinoma of stomach or gastroesophageal junction (34). Later, the usage of apatinib was extended to various cancers, mainly gastric cancer, esophageal cancer, hepatocellular carcinoma, BC, lung cancer, sarcoma, and colorectal cancer (35). Apatinib monotherapy is usually administered at the end of the treatment period or in cases of intolerance to other treatments in patients with ABC. Although its effects are not mentioned in the respective guidelines, it is an alternative for specific patient populations, and its up-to-date clinical data regarding efficacy and safety needs to be collected and provided to clinical workers.

### Efficacy evaluation

#### Response rates to apatinib

A total of six studies were included in the final meta-analysis. In the efficacy analysis, pooled ORR and DCR were found to be 20.4% and 71.6%, respectively. Likewise, apatinib monotherapy was administered to patients with other cancers. The pooled ORRs and DCRs of apatinib monotherapy for refractory lung cancer, liver cancer, gastric cancer, colorectal cancer, and advanced or metastatic osteosarcoma were 20%, 18%, 10%, 13%, and 27%; and 82%, 51%, 66%, 79%, and 64%, respectively (36, 37). Our findings indicate that apatinib monotherapy has a promising ORR and moderate DCR in patients with ABC compared with those in patients with other advanced malignances.

Sunitinib and sorafenib are two other orally delivered antiangiogenic TKIs. In a phase III trial of sunitinib *vs*. capecitabine for patients with HER2-negative ABC, the ORR of 238 patients treated with sunitinib only reached 11% (38). Furthermore, sorafenib showed a lower ORR in a phase II trial of sorafenib alone for 56 patients with MBC, with PR observed in only one patient (2%) (39). On the basis of these data, we believe that apatinib has a superior ORR among orally delivered antiangiogenic TKIs.

Some single-agent drugs are widely used in patients with ABC, including paclitaxel, capecitabine, and gemcitabine. Paclitaxel can generate ORRs of 20.8% and 19.8% and a DCR of 57.9% in previously treated patients with MBC (11, 13). In reviewing prospective studies, each of which enrolled at least 100 previously treated patients with ABC, capecitabine monotherapy had a median ORR of 20% and DCR of 62% among these studies (10). The ORRs by gemcitabine monotherapy was 20% for heavily treated patients with MBC (12). Summarily, apatinib monotherapy has a comparable ORR and DCR to monotherapy of commonly used chemotherapy drugs for previously treated patients with ABC.

Our subgroup analysis revealed that patients with TNBC had a higher ORR than patients with non-TNBC (30.5% and 18.7%, respectively), implying that apatinib can be more effective for a certain subtype of ABC. The difference in ORRs between these subtypes of ABC was also reported in the study by Liu et al., whose main focus was on the efficacy of apatinib with or without other treatment strategies for MBC. Univariate analysis in that study showed that the subtype of patients with MBC was significantly associated with OS (7). Moreover, five of seven patients whose disease control time during apatinib monotherapy was longer than any previously treated regimens were of the TNBC subtype (14). However, the difference shown between the subtypes needs further confirmation with double-arm studies.

The pooled ORR and DCR in patients with a starting dose of apatinib 500 mg/day were 18.6% and 60.9%, respectively, which were lower than patients with a starting dose of 750 mg/day. A potential dose–response relationship was found. However, toxicity associated with the daily 750-mg dose limited regular clinical application, which leaded to a dose interruption rate of 68% (9).

Hu2014 (2) (9) appeared as a source of heterogeneity during the sensitive analysis and, hence, was omitted from the analysis of pooling DCR. Compared with the other included studies in terms of pooling DCR, Hu2014 (2) had a much lower DCR. We believe that this study included too many heavily treated patients, which might have led to a lower DCR since the number of treatment lines before apatinib was a hazard factor for prognosis (33).

#### Impact of apatinib on prognosis

In our study, the pooled median PFS and OS for ABC were 4.00 and 10.43 months, respectively. A phase III trial revealed that apatinib treatment had a median PFS of 2.6 months and a median OS of 6.5 months in patients with advanced or metastatic adenocarcinoma of the stomach or gastroesophageal junction with an initial oral dose of apatinib of 850 mg once daily (34). Moreover, a median PFS of 4.7 months of apatinib treatment at a starting dose of 750 mg was found in patients with advanced non-squamous non–small cell lung cancer following two previous treatment regimens (40). When compared with other orally delivered TKIs for treating ABC, a median PFS of 2.8 months and a median OS of 15.3 months in HER2-negative ABC treated with sunitinib were found in a phase III randomized trial (38). Another orally delivered TKI, sorafenib monotherapy, did not even exhibit treatment activity in patients with MBC who had received prior treatment (41). When comparing with other single-agent chemotherapy drugs, patients with ABC had a median OS of 11.0, 11.0, and 12.8 months by capecitabine (10), gemcitabine (12), and paclitaxel monotherapy, respectively.

These abovementioned results suggest that apatinib has comparable or superior response rates, compared with its effects for patients with other advanced solid malignances, or other orally delivered TKIs or chemotherapy drugs for patients with ABC. To verify the efficacy and safety of apatinib and explore the potential of wide usage in clinic, prospective large-scaled clinical trials are needed. First, apatinib monotherapy needs to be compared with currently widely used drugs, such as paclitaxel and capecitabine, in patients with ABC who failed in previous salvage treatments. Second, comparing the efficacy and safety of apatinib with chemotherapy, endocrine therapy, targeted therapy, or immunotherapy with other guideline-accepted regimens for patients with ABC in different conditions, such as front-line therapy for untreated ABC, or salvage therapy for previously treated ABC. Immunotherapy targeting programmed cell death protein-1 (PD-1) and its ligand (PD-L1) is a well-established therapeutic option for TNBC (42); however, only a low proportion of patients with metastatic TNBC can benefit from PD-1/PD-L1 blockade (43). Combining anti-angiogenesis therapy with PD-1/PD-L1 blockade is a promising combination, for anti-angiogenesis therapy plays an essential role in enhancing the efficacy of immunotherapy (44, 45). Moreover, the efficacy, safety, and drug combination will be explored in other clinical settings, including neoadjuvant therapy and adjuvant therapy. These trials can find out the suitable drugs combination and the patient populations sensitive to apatinib.

### Toxicity evaluation

#### Hematologic AE ≥ grade 3

The most frequently encountered hematologic ≥grade 3 AEs was thrombocytopenia, reaching a proportion of 6.9%. This rate was similar to that reached with sunitinib, another orally delivered antiangiogenic TKI, which leads to 8% grade 3–4 thrombocytopenia (38). Thrombocytopenia was also the most common hematologic ≥ grade 3 AEs when treating bone and soft tissue sarcoma with apatinib, but with a significantly lower pooled proportion of 0.05% (46).

#### Non-hematologic AE ≥ grade 3

The three most frequently encountered non-hematologic ≥grade 3 AEs were hypertension, hand-foot syndrome, and proteinuria, with proportions of 24.3%, 13.4%, and 9.3%, respectively. Similarly, these three AEs were also reported to be the three most common grade 3–4 AEs among solid malignances, with pooled proportions of 7%, 6%, and 4%, respectively (36). These AEs also occurred frequently during the treatment of bone and soft tissue sarcoma with apatinib; however, their proportions ranged from 1.13% to 3.19%, which is much lower than that of AEs during ABC treatment (46).

Regarding the two additional orally delivered antiangiogenic TKIs, sorafenib and sunitinib alone induced a proportion of 4% of patients suffering from grade 3–4 hand-foot skin reaction (39), and 8% and 3.8% of patients suffering from grade 3–4 hand-foot syndrome and hypertension, respectively (38). Apatinib appeared to produce more frequent grade 3–4 AEs during ABC treatment, especially hypertension, compared with its administration for other malignances or the administration of other TKIs for ABC. This may be because of the different tumor characteristics, drug dosages, and number of previous treatment regimens. However, hypertension was considered a protective factor for prolonged PFS in ABC (33). Whether the promising ORR of apatinib treatment for ABC is based on the induced grade 3–4 hypertension remains inconclusive and requires further analysis with a greater number of patients.

In addition, higher toxicity leads to higher rates of dose adjustments. In our meta-analysis, 56.4% of patients experienced treatment interruption, 43.3% underwent dose reduction, and treatment in 12.1% was discontinued. In contrast, only 21% of patients underwent dose reduction during the treatment of advanced or metastatic adenocarcinoma of stomach or gastroesophageal junction whose initial dose of apatinib was 850 mg daily (34). Moreover, only 52% and 28% of patients underwent dosage interruption and dose reduction, respectively, during sunitinib administration for ABC (38).

Our study has several limitations. First, a limited number of studies were included into our final meta-analysis due to the relatively small number of studies focusing on the efficacy of apatinib monotherapy. Consequently, subgroup analysis based on various study designs, such as prospective and retrospective designs, could not be performed. Second, there were discrepancies in the initial doses among the included studies. More specifically, whereas most patients were initially administered with 500 mg of apatinib daily, some patients received 250, 425, 750, or 850 mg of apatinib daily, which may have generated heterogeneities among the results of the studies. Third, although the efficacy and safety of apatinib for ABC were compared with those of apatinib for other advanced malignances or with those of other orally delivered TKIs for ABC, the comparisons were mostly between single-arm studies, and the results need to be further verified by additional controlled studies.

## Conclusions

Our study collected and pooled the data of efficacy and safety of apatinib alone for patients with ABC. This study confirms the reliable efficacy of apatinib monotherapy for ABC. In particular, response rates are comparable or superior for patients with ABC treated with apatinib, compared with apatinib for patients with other advanced solid malignances, or other orally delivered TKIs or some chemotherapy drugs for patients with ABC. However, the non-hematologic grade 3–4 AEs from apatinib for ABC, especially hypertension, are more frequent than other orally delivered TKIs.

## Data availability statement

The original contributions presented in the study are included in the article/**Supplementary Material**. Further inquiries can be directed to the corresponding author.

## Author contributions

XCH contributed to conception and design, studies search, collection and assembly of data, and data analysis and interpretation. XHH contributed to studies search, and data analysis and interpretation. TBY contributed to conception and design, administrative support, collection and assembly of data, and data analysis and interpretation. All authors wrote manuscript and have approved the final version of the manuscript.

## Funding

This work was supported by the research fund of the hospital level from Taizhou People’s Hospital Affiliated to Nanjing Medical University (ZL202001).

## Conflict of interest

The authors declare that the research was conducted in the absence of any commercial or financial relationships that could be construed as a potential conflict of interest.

## Publisher’s note

All claims expressed in this article are solely those of the authors and do not necessarily represent those of their affiliated organizations, or those of the publisher, the editors and the reviewers. Any product that may be evaluated in this article, or claim that may be made by its manufacturer, is not guaranteed or endorsed by the publisher.

## References

[1] Sung H Ferlay J Siegel RL Laversanne M Soerjomataram I Jemal A . Global cancer statistics 2020: GLOBOCAN estimates of incidence and mortality worldwide for 36 cancers in 185 countries. CA Cancer J Clin (2021) 71(3):209–49. doi: 10.3322/caac.21660 33538338

[2] Skinner KE Haiderali A Huang M Schwartzberg LS . Real-world effectiveness outcomes in patients diagnosed with metastatic triple-negative breast cancer. Future Oncol (2021) 17(8):931–42. doi: 10.2217/fon-2020-1021 33207944

[3] Khaled N Bidet Y . New insights into the implication of epigenetic alterations in the EMT of triple negative breast cancer. Cancers (Basel) (2019) 11(4):559. doi: 10.3390/cancers11040559 PMC6521131 31003528

[4] Hanahan D Weinberg RA . The hallmarks of cancer. Cell (2000) 100(1):57–70. doi: 10.1016/s0092-8674(00)81683-9 10647931

[5] Wang X Bove AM Simone G Ma B . Molecular bases of VEGFR-2-Mediated physiological function and pathological role. Front Cell Dev Biol (2020) 8:599281. doi: 10.3389/fcell.2020.599281 33304904 PMC7701214

[6] Li W Man XY Li CM Chen JQ Zhou J Cai SQ . VEGF induces proliferation of human hair follicle dermal papilla cells through VEGFR-2-mediated activation of ERK. Exp Cell Res (2012) 318(14):1633–40. doi: 10.1016/j.yexcr.2012.05.003 22659165

[7] Liu Z Shan J Yu Q Wang X Song X Wang F . Real-world data on apatinib efficacy - results of a retrospective study in metastatic breast cancer patients pretreated with multiline treatment. Front Oncol (2021) 11:643654. doi: 10.3389/fonc.2021.643654 34178630 PMC8224527

[8] Hu X Cao J Hu W Wu C Pan Y Cai L . Multicenter phase II study of apatinib in non-triple-negative metastatic breast cancer. BMC Cancer (2014) 14(1):820. doi: 10.1186/1471-2407-14-820 25376790 PMC4237755

[9] Hu X Zhang J Xu B Jiang Z Ragaz J Tong Z . Multicenter phase II study of apatinib, a novel VEGFR inhibitor in heavily pretreated patients with metastatic triple-negative breast cancer. Int J Cancer (2014) 135(8):1961–9. doi: 10.1002/ijc.28829 24604288

[10] Ershler WB . Capecitabine monotherapy: safe and effective treatment for metastatic breast cancer. Oncologist (2006) 11(4):325–35. doi: 10.1634/theoncologist.11-4-325 16614228

[11] Perez EA Vogel CL Irwin DH Kirshner JJ Patel R . Multicenter phase II trial of weekly paclitaxel in women with metastatic breast cancer. J Clin Oncol (2001) 19(22):4216–23. doi: 10.1200/JCO.2001.19.22.4216 11709565

[12] Rha SY Moon YH Jeung HC Kim YT Sohn JH Yang WI . Gemcitabine monotherapy as salvage chemotherapy in heavily pretreated metastatic breast cancer. Breast Cancer Res Treat (2005) 90(3):215–21. doi: 10.1007/s10549-004-2468-4 15830134

[13] Seidman A Tiersten A Hudis C Gollub M Barrett S Yao T . Phase II trial of paclitaxel by 3-hour infusion as initial and salvage chemotherapy for metastatic breast cancer. J Clin Oncol (1995) 13(10):2575–81. doi: 10.1200/JCO.1995.13.10.2575 7595709

[14] Lü H Zhang M Niu L Zeng H Yan M . Clinical observation of apatinib mesylate for the treatment of multi-drug resistant advanced breast cancer. Natl Med J China (2018) 98(16):1246–9. doi: 10.3760/cma.j.issn.0376-2491.2018.16.012 29747313

[15] Balduzzi S Rucker G Schwarzer G . How to perform a meta-analysis with r: a practical tutorial. Evid Based Ment Health (2019) 22(4):153–60. doi: 10.1136/ebmental-2019-300117 PMC10231495 31563865

[16] Poisot T . The digitize package: extracting numerical data from scatterplots. R J (2011) 3(1):25–6. doi: 10.32614/RJ-2011-004

[17] Pandey S . metaSurvival: Meta-analysis of a single survival curve. In: R package version 0.1.0 (2020). Retrieved from: https://CRAN.R-project.org/package=metaSurvival.

[18] R Core Team . R: A language and environment for statistical computing. In: R foundation for statistical computing. Vienna, Austria (2022).

[19] Tierney JF Stewart LA Ghersi D Burdett S Sydes MR . Practical methods for incorporating summary time-to-event data into meta-analysis. Trials (2007) 8:16. doi: 10.1186/1745-6215-8-16 17555582 PMC1920534

[20] Huang Y Zhao Y Hu X Wu X Li L . Could less apatnib be better? an exploration study of dose-effect of apatnib in 41 stage IV solid tumors. J Clin Oncol (2018) 36(Suppl 15):e24328. doi: 10.1200/JCO.2018.36.15-suppl.e24328

[21] Li H Geng C Zhao H Jiang H Song G Zhang J . Multicenter phase II study of apatinib single or combination therapy in HER2-negative breast cancer involving chest wall metastasis. Chin J Cancer Res (2021) 33(2):243–55. doi: 10.21147/j.issn.1000-9604.2021.02.11 PMC8181870 34158743

[22] Liu G Wang C He Y Mingyan E . Application effect of apatinib in patients with failure of standard treatment for advanced malignant tumours. BMC Pharmacol Toxicology (2019) 20(1):61. doi: 10.1186/s40360-019-0362-2 PMC6819520 31661009

[23] Wang J Chen Y Chen R Wu L Cheng J . Application of apatinib after multifaceted therapies for metastatic breast cancer. Transl Cancer Res (2020) 9(8):4488–97. doi: 10.21037/tcr-19-2588 PMC8797807 35117814

[24] Wang X Wang X Shi Y Wang C Tong Z . Clinical efficacy of apatinib in treating refractory triple-negative advanced breast cancer. Chin J Clin Oncol (2017) 44(15):769–72. doi: 10.3969/j.issn.1000-8179.2017.15.643

[25] Wu X Wang H Wu Y Jin J Zhan Y Zhu G . Efficacy of apatinib on multiple advanced-stage nongastric cancers. J Cancer Res Ther (2019) 15(4):836–41. doi: 10.4103/jcrt.JCRT_24_19 31436240

[26] Shi L Chen Z Wang X . Apatinib to prolong overall survival from recurrence to death in metastatic breast cancer: A single-center experience from east China. J Clin Oncol (2020) 38(Suppl 15):e13090. doi: 10.1200/JCO.2020.38.15-suppl.e13090

[27] Fan M Zhang J Wang Z Wang B Zhang Q Zheng C . Phosphorylated VEGFR2 and hypertension: potential biomarkers to indicate VEGF-dependency of advanced breast cancer in anti-angiogenic therapy. Breast Cancer Res Treat (2014) 143(1):141–51. doi: 10.1007/s10549-013-2793-6 24292957

[28] Zeng T Li W . Efficacy of low-dose apatinib in advanced HER2-negative breast cancer. Ann Oncol (2019) 30:vi114. doi: 10.1093/annonc/mdz338.103

[29] Fan M Hu X Zhang J Wang Z Zhang Q . Hypertension and phosphorylated vascular endothelial growth factor receptor 2 are potential independent predictive factors for progession-free survival in apatinib-treated advanced breast cancer. Cancer Res (2013) 73(Suppl 24):P1–08–26. doi: 10.1158/0008-5472.SABCS13-P1-08-26

[30] Hu XC Zhang J Xu BH Jiang ZF Tong ZS Zhang QY . Multicenter phase II study of apatinib, a novel inhibitor of VEGFR, in heavily pretreated patients with metastatic triple negative breast cancer. Breast (2013) 22:S33. doi: 10.1016/S0960-9776(13)70054-7 24604288

[31] Li H Geng C Song G Jiang H Tong Z Yang J . Apatinib in the treatment of HER-2 negative advanced breast cancer with chest wall metastasis multicenter phase II clinical study. Cancer Res (2020) 80(Suppl 4):P1–19–40. doi: 10.1158/1538-7445.SABCS19-P1-19-40

[32] Lin Y Wu Z Zhang J Hu X Wang Z Wang B . Apatinib for metastatic breast cancer in non-clinical trial setting: Satisfying efficacy regardless of previous anti-angiogenic treatment. Tumour Biol (2017) 39(6):1010428317711033. doi: 10.1177/1010428317711033 28639910

[33] Shen XJ Zhang WJ Ma ZJ Ma MJ Wang LX . Efficacy and safety of apatinib in patients with advanced breast cancer. Chin J Cancer Prev Treat (2019) 26(8):549–53. doi: 10.16073/j.cnki.cjcpt.2019.08.006

[34] Li J Qin S Xu J Xiong J Wu C Bai Y . Randomized, double-blind, placebo-controlled phase III trial of apatinib in patients with chemotherapy-refractory advanced or metastatic adenocarcinoma of the stomach or gastroesophageal junction. J Clin Oncol (2016) 34(13):1448–54. doi: 10.1200/JCO.2015.63.5995 26884585

[35] Tian Z Niu X Yao W . Efficacy and response biomarkers of apatinib in the treatment of malignancies in China: A review. Front Oncol (2021) 11:749083. doi: 10.3389/fonc.2021.749083 34676173 PMC8525889

[36] Sun D Hou H Zhang C Zhang X . The efficacy and safety of apatinib for refractory malignancies: a review and meta-analysis. Onco Targets Ther (2018) 11:6539–54. doi: 10.2147/OTT.S176429 PMC6178936 30323627

[37] Yao H Chen X Tan X . Efficacy and safety of apatinib in the treatment of osteosarcoma: a single-arm meta-analysis among Chinese patients. BMC Cancer (2021) 21(1):449. doi: 10.1186/s12885-021-08154-3 33892656 PMC8063308

[38] Barrios CH Liu MC Lee SC Vanlemmens L Ferrero JM Tabei T . Phase III randomized trial of sunitinib versus capecitabine in patients with previously treated HER2-negative advanced breast cancer. Breast Cancer Res Treat (2010) 121(1):121–31. doi: 10.1007/s10549-010-0788-0 PMC2855860 20339913

[39] Bianchi G Loibl S Zamagni C Salvagni S Raab G Siena S . Phase II multicenter, uncontrolled trial of sorafenib in patients with metastatic breast cancer. Anticancer Drugs (2009) 20(7):616–24. doi: 10.1097/CAD.0b013e32832b2ea0 19739318

[40] Zhang L Shi M Huang C Liu X Xiong JP Chen G . A phase II, multicenter, placebo-controlled trial of apatinib in patients with advanced nonsquamous non-small cell lung cancer (NSCLC) after two previous treatment regimens. J Clin Oncol (2012) 30(15_suppl):7548. doi: 10.1200/jco.2012.30.15_suppl.7548

[41] Moreno-Aspitia A Morton RF Hillman DW Lingle WL Rowland KM Jr. Wiesenfeld M . Phase II trial of sorafenib in patients with metastatic breast cancer previously exposed to anthracyclines or taxanes: North central cancer treatment group and Mayo clinic trial N0336. J Clin Oncol (2009) 27(1):11–5. doi: 10.1200/JCO.2007.15.5242 PMC2645094 19047293

[42] Shadbad MA Safaei S Brunetti O Derakhshani A Lotfinejad P Mokhtarzadeh A . A systematic review on the therapeutic potentiality of PD-L1-Inhibiting MicroRNAs for triple-negative breast cancer: Toward single-cell sequencing-guided biomimetic delivery. Genes (Basel) (2021) 12(8):1206. doi: 10.3390/genes12081206 34440380 PMC8391239

[43] Adams S Schmid P Rugo H Winer E Loirat D Awada A . Pembrolizumab monotherapy for previously treated metastatic triple-negative breast cancer: cohort a of the phase II KEYNOTE-086 study. Ann Oncol (2019) 30(3):397–404. doi: 10.1093/annonc/mdy517 30475950

[44] Chambers A Kundranda M Rao S Mahmoud F Niu J . Anti-angiogenesis revisited: Combination with immunotherapy in solid tumors. Curr Oncol Rep (2021) 23(9):100. doi: 10.1007/s11912-021-01099-7 34269922

[45] Liu J Liu Q Li Y Li Q Su F Yao H . Efficacy and safety of camrelizumab combined with apatinib in advanced triple-negative breast cancer: an open-label phase II trial. J Immunother Cancer (2020) 8(1):e000696. doi: 10.1136/jitc-2020-000696 32448804 PMC7252975

[46] Long Z Huang M Liu K Li M Li J Zhang H . Assessment of efficiency and safety of apatinib in advanced bone and soft tissue sarcomas: A systematic review and meta-analysis. Front Oncol (2021) 11:662318. doi: 10.3389/fonc.2021.662318 33816318 PMC8010174

