# Supplementary material for: Efficacy and safety of apatinib monotherapy for patients with advanced breast cancer: a systematic review and meta-analysis
Source: Front Oncol. 2022 Aug 1;12:940171. doi: 10.3389/fonc.2022.940171 (PMC9376484; doi:10.3389/fonc.2022.940171)

Supplementary Material

## Supplementary Figures

**Supplementary Figure 1:** A workflow diagram showing the main study steps.


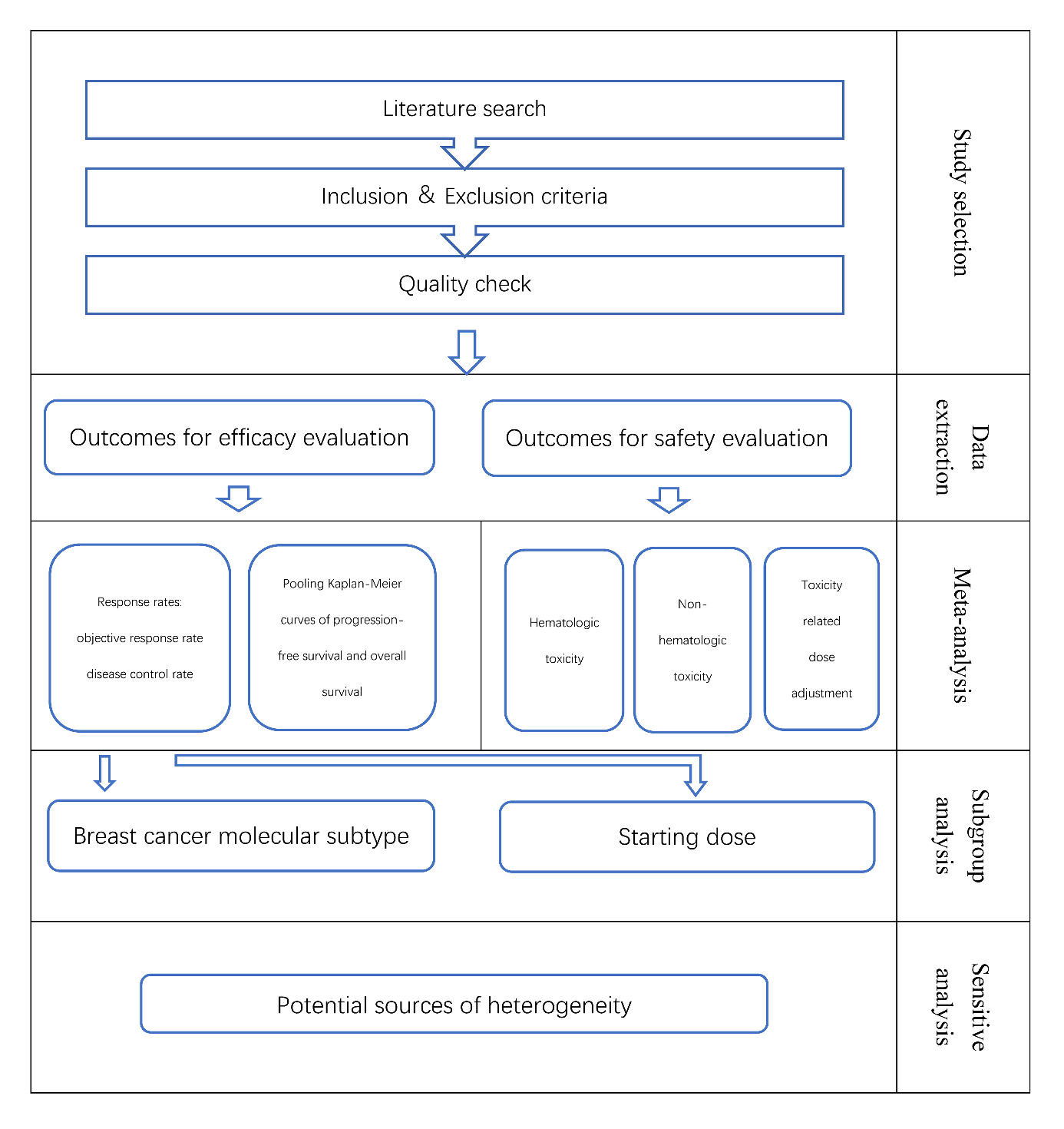


**Supplementary Figure 2:** Meta-analysis of objective response rates of patients initially administered with 500 mg apatinib daily.


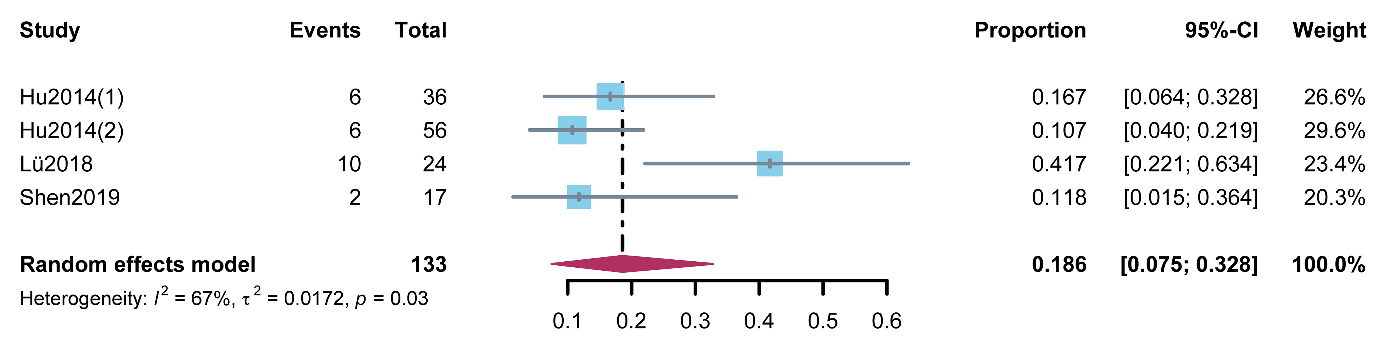


**Supplementary Figure 3:** Meta-analysis of disease control rates of patients initially administered with 500 mg apatinib daily.


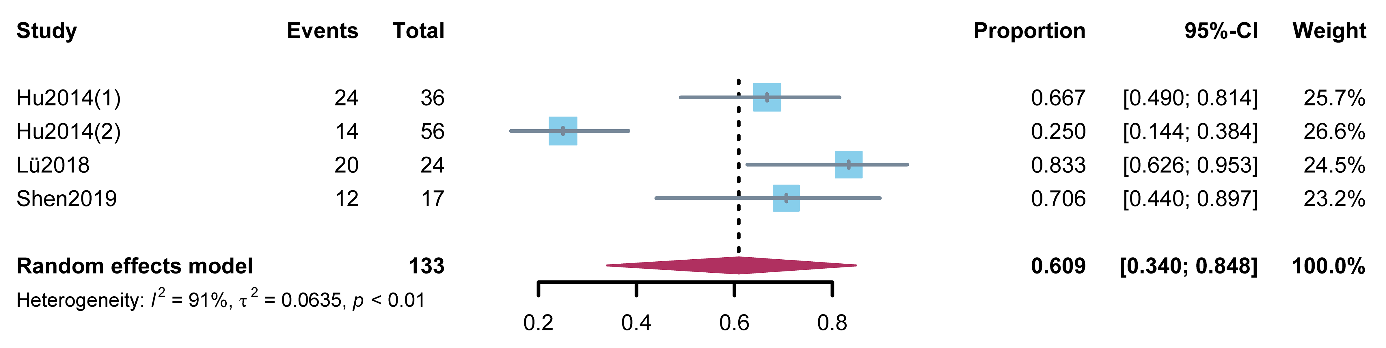


**
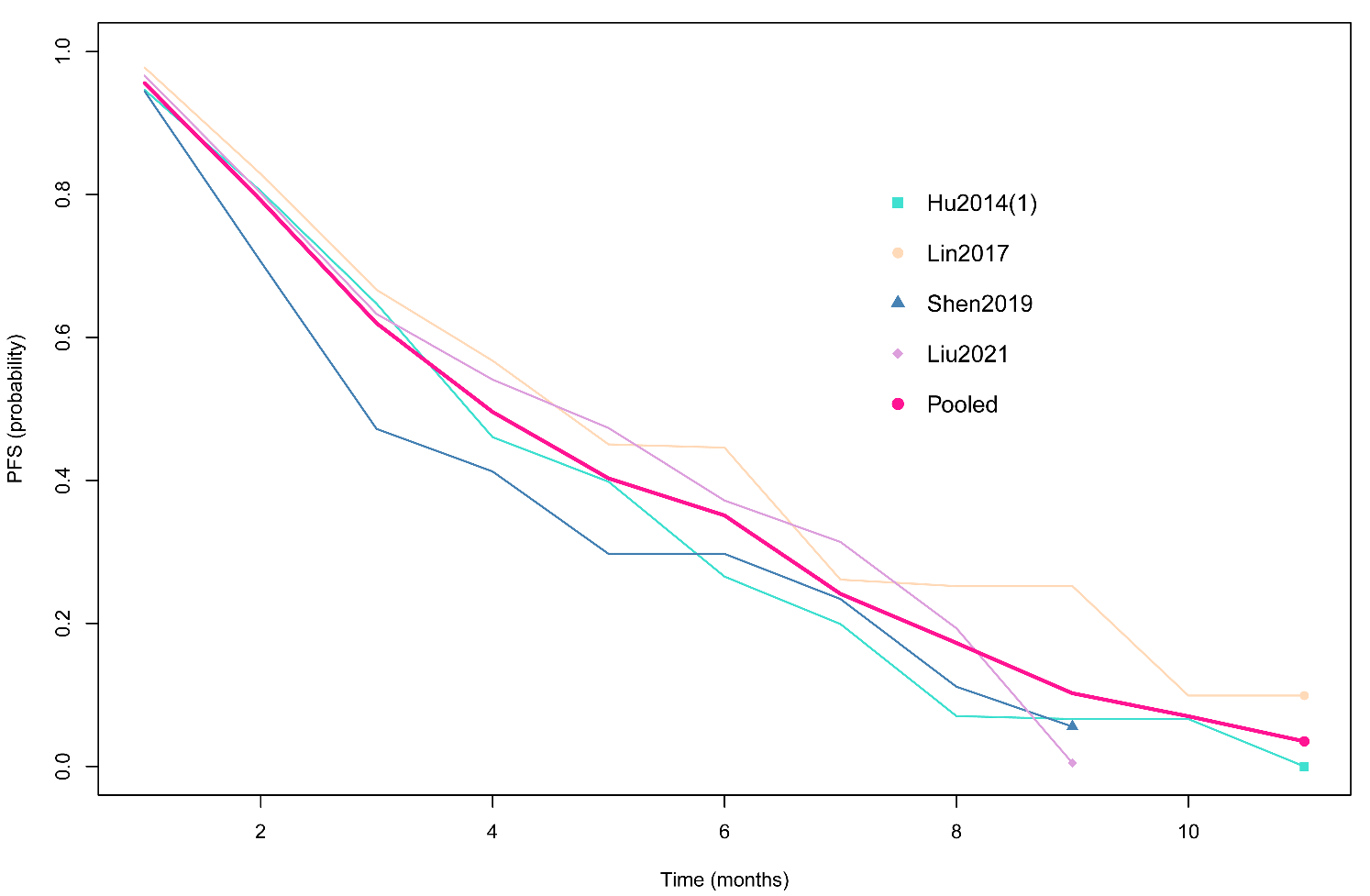
Supplementary Figure 4.** Pooled Kaplan-Meier curve of progression-free survival. PFS, progression-free survival.

**Supplementary Figure 5.** Pooled Kaplan Meier curve of overall survival. **OS**, overall survival.


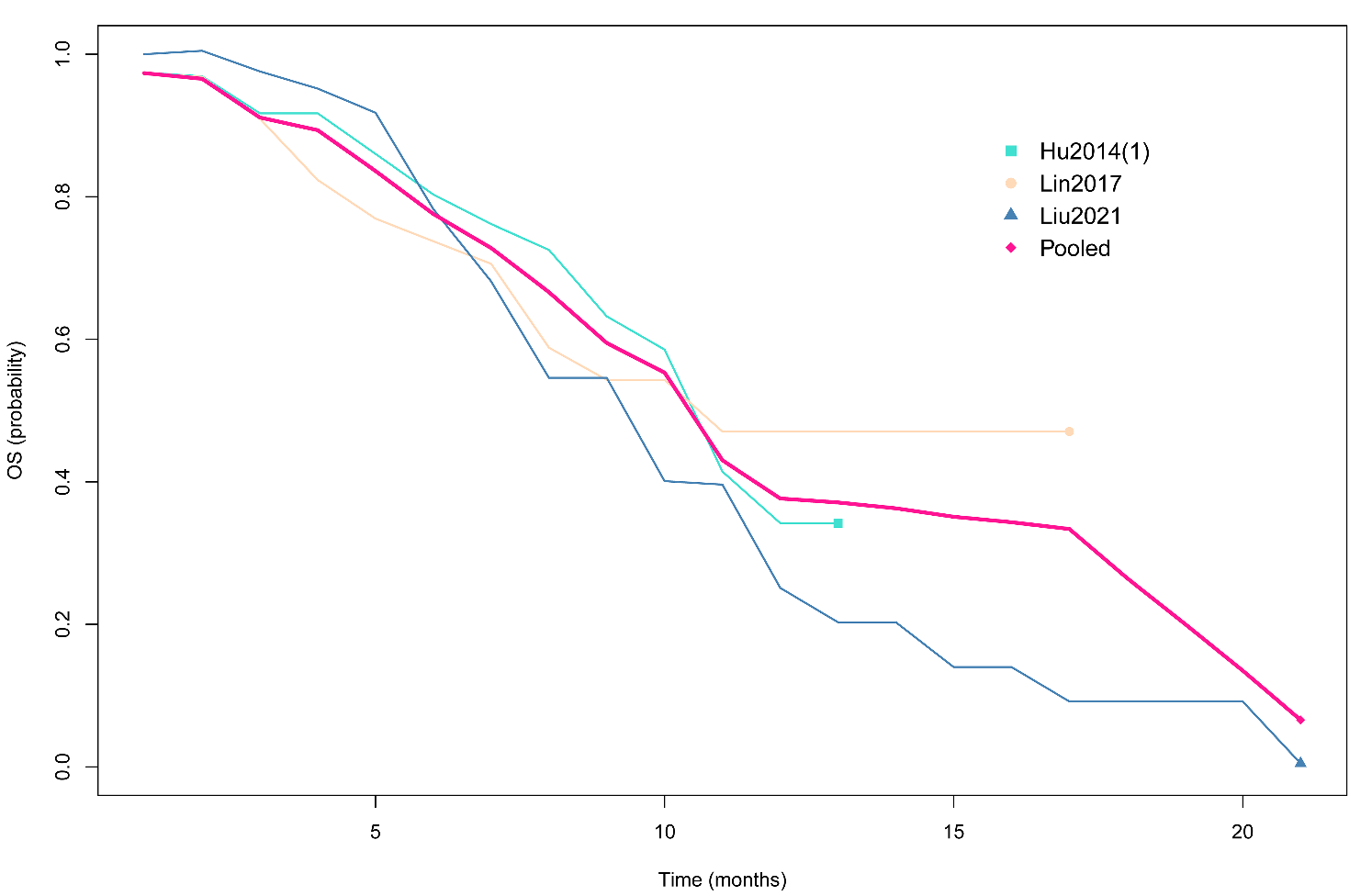

Supplement: Supplementary file 1 [file DataSheet_1.docx]
